# Adherence to Mediterranean diet and physical activity practice in general population: an intersectional analysis of inequalities by sex and economic status

**DOI:** 10.3389/fpubh.2025.1727223

**Published:** 2025-12-15

**Authors:** Glòria Reig-García, Joan Martínez-Sancho, Alícia Baltasar-Bagué, Glòria Mateu-Figueras, Maria Buxó, Ruth Martí-Lluch, Lluís Zacarías-Pons, Josep Puig, Rafel Ramos-Blanes, José-Manuel Fernández-Real, Josep Garre-Olmo

**Affiliations:** 1 Department of Nursing, University of Girona, Girona, Catalonia, Spain; 2 Statistical Unit, Girona Biomedical Research Institute (IDIBGI), Salt, Catalonia, Spain; 3 Department of Computer Science, Applied Mathematics and Statistics, University of Girona, Girona, Catalonia, Spain; 4 Vascular Health Research Group of Girona, Institut Universitari per a la Recerca a l’Atenció Primària Jordi Gol i Gurina (IDIAPJGol), Girona, Spain; 5 Parc Hospitalari Martí Julià, Institut d'Investigació Biomèdica de Girona (IDIBGI), Salt, Spain; 6 Department of Medical Sciences, School of Medicine, Campus Salut, Universitat de Girona, Girona, Spain; 7 Network for Research on Chronicity, Primary Care, and Health Promotion (RICAPPS), Madrid, Spain; 8 Radiology Department CDI and IDIBAPS, Hospital Clinic, Barcelona, Spain; 9 Atenció Primària, Institut Català de la Salut, Girona, Catalonia, Spain; 10 Department of Diabetes, Endocrinology and Nutrition, Dr. Josep Trueta University Hospital, Girona, Catalonia, Spain; 11 Nutrition, Eumetabolism and Health Group, Girona Biomedical Research Institute (IDIBGI), Girona, Catalonia, Spain; 12 CIBER Centro de Investigación Biomédica en Red de Fisiopatología de la Obesidad y Nutrición (CIBERobn), Madrid, Spain

**Keywords:** economic status, health inequalities, Mediterranean diet, physical activity, sex

## Abstract

**Background:**

Inequalities in physical activity (PA) practice and adherence to Mediterranean diet (MD) between sex or socioeconomic groups have been described. We analyzed inequalities using an intersectional approach combining sex and economic difficulties (ED) in a population-based adult sample from Girona (Spain).

**Methods:**

Cross-sectional study including 1,425 inhabitants. ED were assessed with a self-reported question on perceived ability to make ends meet and dichotomized (with/without ED). Sex and ED were employed to form four intersectional positions (men/women × with/without ED). PA was assessed using the Minnesota-Regicor questionnaire, total weekly metabolic equivalents of task were calculated, and PA was dichotomized as low (first quartile) and not low. Adherence to MD was measured with the PREDIMED questionnaire and dichotomized as non-adherence/adherence. Sociodemographic and lifestyle characteristics were registered. We estimated crude and adjusted prevalence differences in low PA practice and non-adherence to MD corresponding to joint, referent, and excess intersectional inequalities using linear binomial regression models with identity link, which directly estimate prevalence differences in percentage points (pp). The discriminatory accuracy of the models was assessed using the area under the receiver operating characteristic curve (AUC-ROC).

**Results:**

The prevalence of low PA was higher among women and increased by the presence of ED (26.7% vs. 40.9%). Overall, 27.8% reported ED and the four intersectional positions ranged from 10.5 to 40.3%. The adjusted joint disparity in low PA between women with ED (doubly disadvantaged) and men without (doubly advantaged) was 20.8 pp, of which 11.6 pp represented excess intersectional inequality. For non-adherence to MD, only the referent disparity for sex was statistically significant (8.54 pp). The AUC-ROC ranged from 0.62 for low PA to 0.67 for non-adherence to MD, with small increases when adding the intersectional groups.

**Conclusion:**

In this study, inequalities in adherence to MD were modest, mainly by sex and with low discriminatory accuracy, suggesting that population-wide interventions, complemented by attention to sex differences, may be more appropriate than targeted strategies. In contrast, low PA was markedly higher in women with ED than in men without. This highlights the importance of intersectional inequalities in PA and the need for universal policies that also prioritize socioeconomically disadvantaged women.

## 1 Introduction

Adherence to physical activity (PA) practice and a healthy diet pattern, such as the Mediterranean diet (MD), are key determinants of overall health, chronic disease prevention and reduced mortality (1, 2). Robust evidence shows that being active and following a MD are associated with lower risk of cardiovascular diseases, hypertension, diabetes and some types of cancer (3). In addition, this lifestyle pattern has a positive effect on people’s mental health and overall wellbeing (4). These benefits are thought to be mediated, at least in part, through improvements in cardiometabolic regulation, reductions in low-grade inflammation and favorable effects on the gut microbiota (5).

PA is generally understood as any bodily movement produced by skeletal muscles that requires energy expenditure, encompassing activities performed during leisure time, domestic tasks and work (6). The MD is a predominantly plant-based dietary pattern characterized by high consumption of vegetables, fruits, cereals, nuts, and legumes, most of them cooked by adding substantial amounts of olive oil, with moderate usage of fish, seafood or dairy, and limited intake of meat and alcohol (mostly red wine) (7). In recognition of the strong link between PA and health (8), the 2020 WHO global guidelines reaffirm that investment in PA continues to be a best buy for public health (6), and encourage countries to implement programs and policies to meet the guidelines targets. On the other hand, the MD, one of the most studied and well-known dietary patterns worldwide, has been associated with a wide range of benefits for health (2). Both behaviors have been identified by the WHO as key components in a lifestyle for a healthy aging (9).

However, PA and MD are not equally distributed across social groups. Global surveillance from 358 surveys across 168 countries, including 1.9 million participants, showed that in 2016 the global prevalence of insufficient PA remained high (27.5%), with women having an 8 percentage-point higher prevalence of insufficient PA than men, and these rates remained stable over the past decade (10). Other studies have consistently revealed that, in most countries, women were less active than men (11). Likewise, income inequality has a differential effect on levels of PA in high- and middle-income countries, compared to low income countries, caused by factors, such as infrastructures and access to facilities or resources between countries (12). These findings point to marked sex and economic inequalities in PA, but they are typically examined along a single social axis.

Similar patterns of inequality have been described for adherence to the MD. The studies on sex differences suggest that women are more likely to adhere to the MD (13), although it has been pointed that sex differences in adherence prevalence are related to both geographic location and socioeconomic status of the country (14). This may be due to cultural factors that promote healthy eating habits among women, as well as differences in taste preferences and food choices between sex groups (15). Regarding income, some studies have found that people with higher economic incomes are more likely to adhere to the MD (13). Factors such as access to fresh products, availability of healthy food options, and cultural norms around food influence dietary habits among different income groups (16).

Most of the evidence summarized has examined sex or economic status separately. There is still a limited number of population-based studies that analyze how these axes jointly shape inequalities in PA and MD adherence within an explicit intersectional framework. Intersectionality theory emphasizes that social positions such as sex and economic status do not operate independently, but interact to generate specific configurations of advantage and disadvantage that may reveal inequalities that remain hidden in single-axis analyses. To address this gap, we conducted a population-based study among adults aged 18 years and older living in the province of Girona (Catalonia, Spain). Using an intersectional approach, our aims were to estimate population-based inequalities in PA practice and adherence to the MD between groups defined by the intersection of sex and economic difficulties (ED), decomposing the joint inequality into sex-related, economic-related and excess intersectional components (17) and to assess the additional discriminatory accuracy contributed by the intersectional groups beyond each axis considered separately.

## 2 Methods

### 2.1 Study design and settings

This was a cross-sectional study that used data from the Girona Healthy Region Study (GHR Study), which is a population-based observational study aimed to identify healthy lifestyle determinants in the general population aged 18 years and over living in the province of Girona (Catalonia, Spain). The province includes both urban and rural municipalities, with a large number of small villages and a limited number of cities with more than 10,000 inhabitants. The study protocol of the GHR Study was approved by the Ethics Committee of the IDIAPJGol (ref. 20/041-P).

### 2.2 Participants and sampling

**Figure 1:**
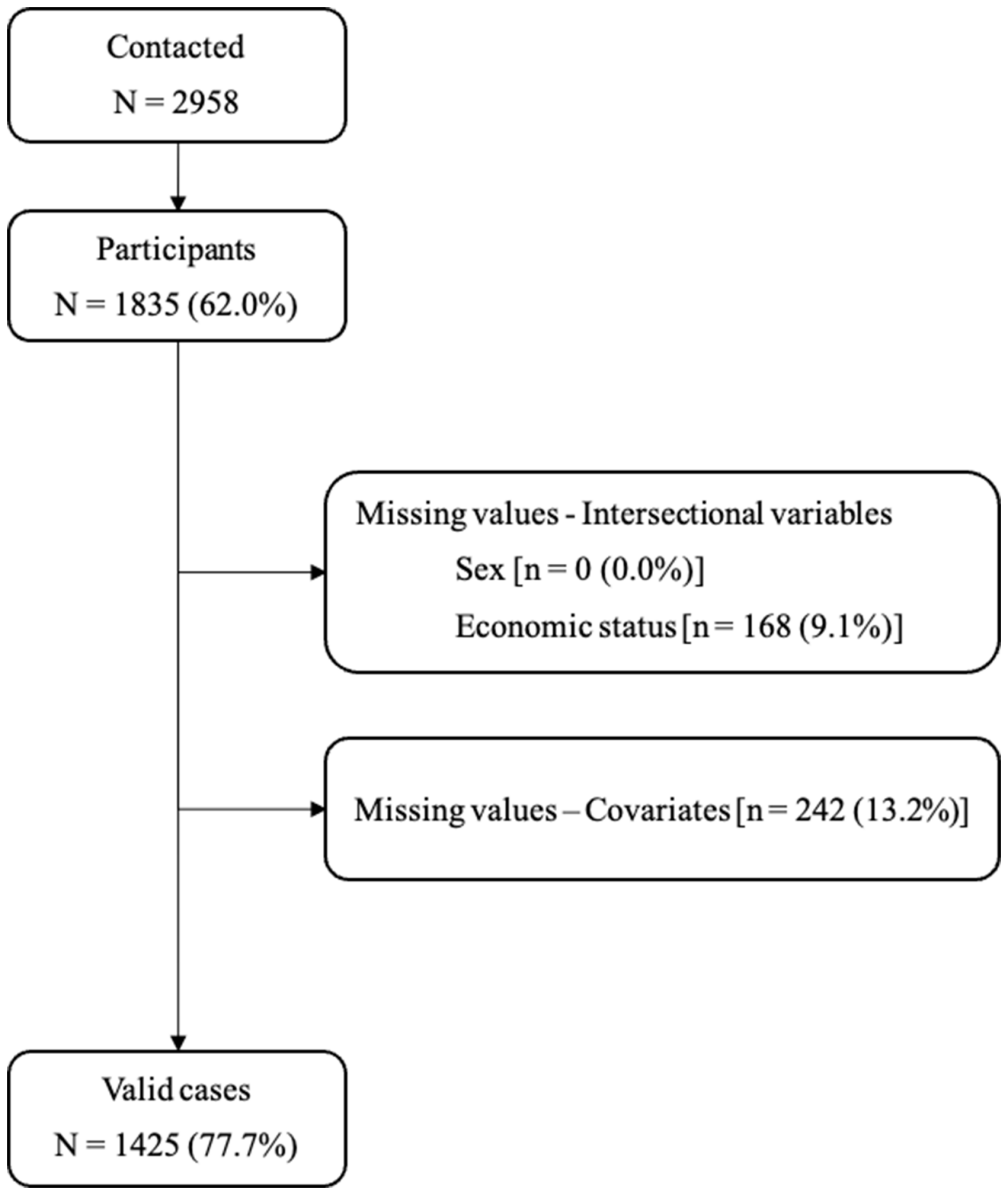
Study participation flow diagram.

Participants were selected using a cluster and stratified random sampling design. Municipalities in the province of Girona were used as first-stage sampling units and were stratified according to population size, distinguishing rural municipalities (≤10,000 inhabitants) from urban municipalities (>10,000 inhabitants). Within each stratum, a random sample of adults aged 18 years and older was drawn from the population assigned to primary care centers in the selected municipalities with sample sizes in each stratum proportional to the population. The inclusion criteria included age equal or above 18 years, and signature of the informed consent. Subjects with terminal illness, cognitive impairment or dementia, intellectual disability, or being institutionalized were excluded. The fieldwork of the GRS Study started on February 22, 2019, and was temporally interrupted on January 30, 2020, due to the state of alarm declared by the Spanish government on March 15, 2020 due to the onset of the COVID-19 pandemic. The study was resumed on September 20, 2021 and study participants’ recruitment was finished on May 5, 2023. 2,958 individuals were contacted and invited to participate, of which 1,835 agreed to participate in the GRS Study (response rate of 62.0%). For this study we used valid cases with complete information on the intersectional variables and covariates. The overall proportion of missing data in these variables was relatively low, as shown in Figure 1. To assess the potential impact of exclusions, we compared key characteristics (age, sex, economic difficulties, low PA and non-adherence to the MD) between included and excluded participants. These comparisons did not reveal meaningful differences, suggesting that selection bias due to missing data is likely to be limited (Supplementary Table 1).

### 2.3 Data collection and measures

The data collection was performed in the Primary Care settings of the study participants’ towns. Participants were informed by postal mail and subsequently received a telephone call to schedule one standardized visit lasting 1.5 h. Trained nurses administered a structured interview covering sociodemographic characteristics, household composition, employment, perceived economic difficulties and health-related behaviors (smoking, alcohol consumption, sedentary time), followed by questionnaires on Mediterranean diet adherence and physical activity. Participants underwent an anthropometric examination including weight, height, and waist circumference measurements using a flat platform (SECA 635), a roll-up measuring tape (SECA 206), and an ergonomic circumference measuring tape (SECA 201).

The intersectional variables were sex and economic difficulties. Sex was measured using the question “*Which sex did you have when you were born*?” and was categorized as male or female. Economic difficulties were operationalized as a binary subjective measure of financial strain using the Deleeck “making ends meet” approach (18). Participants were asked: “*Thinking of your household’s total monthly income, would you say that your household is able to make ends meet…?*” with four response options (with great difficulty, with some difficulty, fairly easily, easily). Responses “with great difficulty” and “with some difficulty” were coded as having economic difficulties, whereas “fairly easily” and “easily” were coded as relative financial ease. This indicator captures perceived economic strain, is closely related to material deprivation, and is widely used in European poverty research as a simple proxy of economic hardship when detailed income or expenditure data are not available (19).

The outcome variables were the adherence to MD and the degree of PA. Adherence to MD was measured using the PREDIMED questionnaire (17). This questionnaire includes 14 questions to assess the frequency of food consumption (olive oil, vegetables, fruit, red meat, animal fats, carbonated beverages, red wine, fish/seafood, nuts, commercial food, traditional dishes) and eating habits (preferred cooking fat used and meat consumed). Each question has a possible score of 0 or 1 and the total score ranges between 0 and 14 points. We dichotomized the score into 0–5 (non-adherence) and 6 or above (adherence, including moderate and high). PA was measured using the Minnesota-Regicor Short Physical Activity Questionnaire for the Adult Population. This questionnaire includes questions to collect information on the type of six activities (walking, brisk walking, gardening, walking trails, climbing stairs, and sport activities), the frequency (days/week), and the duration (minutes/day). Using this information, we calculated the metabolic equivalents of task (METs) multiplying the intensity (light, moderate and vigorous) of each activity and the duration spent on each activity. We dichotomized the PA practice as low (first quartile) or not low (above the first quartile) (20).

For the current study, we employed the following covariates: age (in years), marital status (married/cohabiting, unmarried/not cohabiting), level of education (primary, secondary, tertiary), the count of individuals residing in the participants’ households, the count of household members requiring assistance due to limitations in activities of daily living, the number of dependents in the household under 16 years of age, employment status (working, not working), workday arrangement (single shift, split shift, other), habits related to tobacco consumption (current, former, never) and alcohol consumption past 7 days (yes/no), body mass index categories (underweight [<18.5 kg/m (2)], normal weight [18.50–24.99 kg/m^2^], overweight [25.0–29.99 kg/m^2^], obesity [≥30 kg/m^2^]), and the hours of sedentary activities, such as watching television, using a computer, or playing videogames, on non-working days.

### 2.4 Intersectional groups by sex and economic difficulties

We constructed an intersectional variable by cross-classifying sex and economic difficulties, yielding four mutually exclusive groups. For each outcome, we focused on the adverse event (low PA or non-adherence to the MD) and defined “advantaged” categories as those expected to have a lower prevalence of the adverse outcome, based on previous evidence and our theoretical framework on intersectional inequalities. For low physical activity, prior literature shows that men and individuals without economic difficulties tend to have lower risk of low PA (12) and we defined the four cells of the 2 × 2 table as *μ*₀₀ (men without economic difficulties—advantaged on both axes), *μ*₀₁ (men with economic difficulties—advantaged by sex, disadvantaged economically), *μ*₁₀ (women without economic difficulties—disadvantaged by sex, advantaged economically), and *μ*₁₁ (women with economic difficulties—disadvantaged on both axes). For non-adherence to the Mediterranean diet, previous studies generally indicate that women and higher economic position are associated with better adherence to the MD (13) and we defined the groups as *μ*₀₀ (women without economic difficulties—advantaged on both axes), *μ*₀₁ (women with economic difficulties—disadvantaged economically), *μ*₁₀ (men without economic difficulties—disadvantaged by sex), and *μ*₁₁ (men with economic difficulties—disadvantaged on both axes).

### 2.5 Statistical analysis

Categorical variables were expressed as absolute and relative frequencies and continuous variables as means and standard deviations. A bivariate analysis was performed to determine which variables were associated with the two outcomes (PA and MD adherence). Categorical variables were compared using the chi-square test or Fisher exact test, where appropriate. Continuous variables were analyzed using either Student’s t test or Mann–Whitney U test. These comparisons were descriptive, and *p* values were not adjusted for multiple testing.

For each outcome, inequalities between the four intersectional positions were performed using the methodology described by Jackson et al. (21), with the doubly advantaged position as the reference group. In both outcomes, *μ*₀₀ represents the doubly advantaged reference group and *μ*₁₁ the doubly disadvantaged group, whereas *μ*₀₁ and *μ*₁₀ are singly disadvantaged positions. Following the additive decomposition proposed by Jackson, the joint inequality was defined as the prevalence difference between *μ*₁₁ and *μ*₀₀ (*μ*₁₁ − *μ*₀₀). The referent inequalities for sex and economic difficulties were defined as *μ*₁₀ − *μ*₀₀ and *μ*₀₁ − *μ*₀₀, respectively. The excess intersectional inequality was obtained as the difference between the joint and the sum of the referent inequalities, that is *μ*₁₁ − *μ*₀₀ − (*μ*₁₀ − *μ*₀₀) − (*μ*₀₁ − *μ*₀₀) = *μ*₁₁ − *μ*₁₀ − *μ*₀₁ + *μ*₀₀. All inequalities were expressed as prevalence differences in percentage points. A generalized linear model with a binomial family distribution specified for the outcome, and an identity link function was used to estimate the joint and referent inequalities. Each inequality was expressed as prevalence differences, in percentage points (pp.) with 95% confidence interval. Two models were built for each of the outcome variables, a crude model only including the intersectional variable and a multivariate adjusted model for a set of sociodemographic, household, employment and lifestyle characteristics. These variables were selected *a priori* as potentially relevant determinants of PA and MD that may also vary across intersectional positions, and only those showing statistically significant bivariate associations (*p* < 0.05) with each outcome were retained. Some of these covariates may lie on the causal pathway between economic difficulties and PA/MD and the adjusted estimates are not intended to represent controlled direct effects, but rather intersectional inequalities after accounting for compositional differences in these characteristics. Crude and adjusted results are presented side by side to allow readers to assess the robustness of the inequalities to covariate adjustment.

In addition to estimating prevalence differences between intersectional groups, we assessed the discriminatory accuracy (DA) of the models, understood as the ability to distinguish individuals with and without the outcome based on their characteristics. DA does not quantify causal effects or inequalities per se, but rather the extent to which risk is concentrated within particular groups. We used the area under the receiver operating characteristic curve (AUC-ROC) as a global measure of DA. We fitted three nested logistic regression models for each outcome. Model 1 included only statistically significant covariates for each outcome, model 2 added sex and economic difficulties as separate predictors, and model 3 further added the four intersectional positions (sex × economic difficulties). Comparing AUC-ROC values across these models allowed us to evaluate the incremental contribution of sex and economic difficulties beyond other covariates, and the intersectional groups beyond the two single axes. AUC values range from 0.5 (no discriminatory capacity) to 1.0 (perfect discrimination). In this study, we interpreted AUC values around 0.6–0.7 as indicating low-to-moderate discriminatory accuracy and considered changes in AUC of less than 0.05 to be small and of limited substantive importance.

Data analyses were performed under the valid-cases approach. We did not formally model the missingness mechanism nor apply multiple imputation, because the amount of missing data was modest and multiple imputation would have required additional modeling assumptions for several binary and derived variables (including the intersectional strata and outcomes), with limited expected benefit in terms of precision. Therefore, we considered complete-case analysis to be a reasonable and transparent primary approach for this cross-sectional study.

RStudio 4.3.0 with the Compare Groups package was used for the descriptive and bivariate analyses. Stata 16.1 was used to perform regression analyses. All *p* values were 2-sided, and *p* less than 0.05 were considered statistically significant.

## 3 Results

In the final analytical sample (*n* = 1,425), the mean age of participants was 50.0 years (SD = 16.8; range = 18–95). Overall, 57.6% were women and 64.4% were married or cohabiting with others. Regarding the participants’ household characteristics, the median number of persons in the household was 3 (Interquartile range = 2–4), 34.2% included individuals under 16 years, and 9.3% included persons that required support for the activities of daily living. Regarding the education level, 24.5% completed primary school, 52.3% attained high school and 23.2% achieved a university degree, and 77.1% had an employment. The prevalence of overweight and obesity was 59.5%, current smokers represented 19.6, and 57.6% consumed alcohol during the past 7 days. The mean hours of sedentary activities during non-working days were 3.3 (SD = 2.1).

Overall, 27.8% of participants reported great or some difficulty in making ends meet. Women without economic difficulties accounted for 40.3% of participants, men without economic difficulties for 31.9%, and men and women with economic difficulties for 10.5 and 17.3%, respectively. 168 individuals were excluded due to missing data on economic difficulties, as shown in Figure 1. Table 1 reports the sample characteristics stratified by these four intersectional groups. Except for the number of persons in the household, statistically significant differences were observed between intersectional groups across all covariates. Participants with economic difficulties showed a more disadvantaged profile. For example, tertiary education ranged from 30.1% in women without economic difficulties to 10.0% in men with economic difficulties, the prevalence of overweight or obesity (BMI ≥ 25) ranged from 50.0% in women without economic difficulties to 72.0% in men with economic difficulties, and current smoking ranged from 16.4% in women without economic difficulties to 30.7% in men with economic difficulties.

**Table 1:** Study participants characteristics by intersectional groups.

Variables	Male/No economic difficulties (*n* = 454; 31.9%)	Male/Economic difficulties (*n* = 150; 10.5%)	Female/No economic difficulties (*n* = 574; 40.3%)	Female/Economic difficulties (*n* = 247; 17.3%)	*p*
Age, mean (SD)	51.6 (16.8)	48.5 (15.9)	50.1 (17.5)	47.6 (15.7)	0.017
Marital status, *n* (%)					<0.001
Married/cohabiting	317 (69.8)	96 (64.0)	379 (66.0)	125 (50.6)	
Unmarried/not cohabiting	137 (30.2)	54 (36.0)	195 (34.0)	122 (49.4)	
Education level, *n* (%)					<0.001
Primary	113 (24.9)	48 (32.0)	119 (20.7)	69 (27.9)	
Secondary	238 (52.4)	87 (58.0)	282 (49.1)	138 (55.9)	
Tertiary	103 (22.7)	15 (10.0)	173 (30.1)	40 (16.2)	
Persons/household, median (IQR)	3 (2–4)	3 (2–4)	3 (2–4)	3 (2–4)	0.326
Persons with ADL support, *n* (%)	29 (6.39)	15 (10.0)	54 (9.41)	34 (13.8)	0.015
Persons <16 years, *n* (%)	145 (31.9)	57 (38.0)	180 (31.4)	106 (42.9)	0.006
Workday at job, *n* (%)					<0.001
Single shift	90 (19.8)	26 (17.3)	162 (28.2)	59 (23.9)	
Split shift	132 (29.1)	30 (20.0)	129 (22.5)	38 (15.4)	
Other	142 (31.3)	50 (33.3)	163 (28.4)	78 (31.6)	
Not working	90 (19.8)	44 (29.3)	120 (20.9)	72 (29.1)	
Overweight/Obesity (BMI > 25), *n* (%)	293 (64.5)	108 (72.0)	287 (50.0)	160 (64.8)	<0.001
Tobacco consumption, *n* (%)					<0.001
Never smoker	219 (48.2)	54 (36.0)	373 (65.0)	146 (59.1)	
Former smoker	148 (32.6)	50 (33.3)	107 (18.6)	48 (19.4)	
Current smoker	87 (19.2)	46 (30.7)	94 (16.4)	53 (21.5)	
Alcohol within past 7 days, *n* (%)	325 (71.6)	91 (60.7)	307 (53.5)	98 (39.7)	<0.001
Hours/non-working day TV/computer/videogames, mean (SD)	3.39 (2.31)	4.08 (2.20)	3.15 (1.68)	3.24 (2.11)	<0.001

In descriptive bivariate analysis, sociodemographic and lifestyle characteristics varied between individuals above the second quartile of PA practice and those below. Participants in the first quartile (*n* = 356) tended to be slightly older, less educated, and with a higher frequency of persons living in the household that required support for the activities of daily living than participants in the second to fourth quartiles (*n* = 1,069). Furthermore, overweight and obesity were more common, as was tobacco consumption. Additionally, they reported spending more hours engaged in sedentary activities during non-working days. However, alcohol consumption in past 7 days was lower (Table 2). Likewise, participants with non-adherence to the MD (*n* = 459) exhibited differences in sociodemographic and lifestyle characteristics than those with moderate or high adherence (*n* = 966). Specifically, they tended to be younger, more often unmarried, lived with a larger number of household members, including those under 16 years. Additionally, they were more likely to be current smokers and reported spending more hours in sedentary activities during non-working days (Table 3).

**Table 2:** Study participants characteristics by physical activity practice.

Variables	METs 2nd–4th quartiles (*n* = 1,069; 67.8%)	METs 1st quartile (*n* = 356; 25.0%)	*p*
Age, mean (SD)	49.3 (16.8)	52.2 (16.8)	0.005
Marital status, *n* (%)			0.958
Married/cohabiting	687 (64.3)	230 (64.6)	
Unmarried/not cohabiting	382 (35.7)	126 (35.4)	
Education level, *n* (%)			0.001
Primary	231 (21.6)	118 (33.1)	
Secondary	571 (53.4%)	174 (48.9)	
Tertiary	267 (25.0)	64 (18.0)	
Persons/household, median (IQR)	3 (2–4)	3 (2–4)	0.069
Persons with ADL support, *n* (%)	82 (7.67)	50 (14.0)	<0.001
Persons <16 years, *n* (%)	355 (33.2)	133 (37.4)	0.172
Workday at job, *n* (%)			0.104
Single shift	249 (23.3)	88 (24.7)	
Split shift	262 (24.5)	67 (18.8)	
Other	325 (30.4)	108 (30.3)	
Not working	233 (21.8)	93 (26.1)	
Overweight/Obesity (BMI > 25), *n* (%)	611 (57.2)	237 (66.6)	0.002
Tobacco, *n* (%)			0.020
Never smoker	590 (55.2)	202 (56.7)	
Former smoker	282 (26.4)	71 (19.9)	
Current smoker	197 (18.4)	83 (23.3)	
Alcohol within past 7 days, *n* (%)	650 (60.8)	171 (48.0)	<0.001
Hours/non-working day TV/computer/videogames, mean (SD)	3.25 (1.91)	3.61 (2.39)	0.009

**Table 3:** Study participants characteristics by adherence to Mediterranean diet.

Variables	Moderate/high MD adherence (*n* = 966; 67.8%)	No MD adherence (*n* = 459; 31.2%)	*p*
Age, mean (SD)	52.6 (16.8)	44.4 (15.5)	<0.001
Marital status, *n* (%)			0.007
Married/cohabiting	645 (66.8)	272 (59.3)	
Unmarried/not cohabiting	321 (33.2)	187 (40.7)	
Education level, *n* (%)			0.580
Primary	240 (24.8)	109 (23.7)	
Secondary	496 (51.3)	249 (54.2)	
Tertiary	230 (23.8)	101 (22.0)	
Persons/household, median (IQR)	3 (2–4)	3 (2–4)	<0.001
Persons with ADL support, *n* (%)	95 (9.83)	37 (8.06)	0.326
Persons <16 years, *n* (%)	304 (31.5)	184 (40.1)	0.002
Workday at job, *n* (%)			0.202
Single shift	225 (23.3)	112 (24.4)	
Split shift	211 (21.8)	118 (25.7)	
Other	296 (30.6)	137 (29.8)	
Not working	234 (24.2)	92 (20.0)	
Overweight/Obesity (BMI > 25), *n* (%)	574 (59.4)	274 (59.7)	0.967
Tobacco, *n* (%)			<0.001
Never smoker	544 (56.3)	248 (54.0)	
Former smoker	263 (27.2)	90 (19.6)	
Current smoker	159 (16.5)	121 (26.4)	
Alcohol within past 7 days, *n* (%)	566 (58.6)	255 (55.6)	0.305
Hours/non-working day TV/computer/videogames, mean (SD)	3.22 (1.92)	3.60 (2.27)	0.002

**Figure 2:**
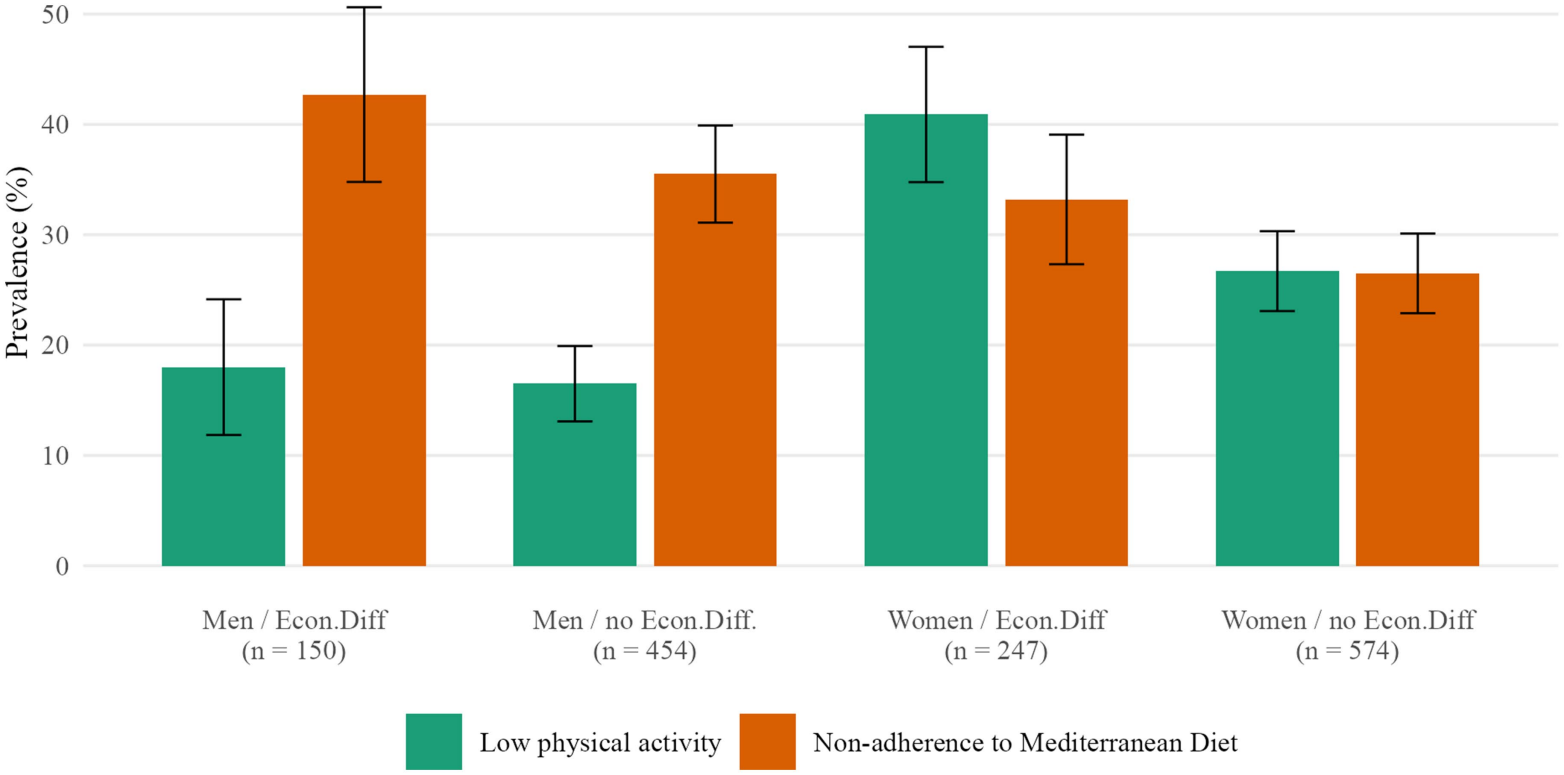
Crude prevalence of non-adherence to MD and low physical activity by intersectional group by sex and economic difficulties.

Crude prevalences of the outcomes across the four intersectional positions are shown in Figure 2. For low PA, the prevalence was 16.5% for men without economic difficulties (*μ*₀₀, doubly advantaged) and 18.0% for men with economic difficulties (*μ*₀₁). Among women, the corresponding prevalences were 26.7% for those without economic difficulties (*μ*₁₀) and 40.9% for those with economic difficulties (*μ*₁₁, doubly disadvantaged). Thus, low PA was more frequent in women than in men, and particularly concentrated among women with economic difficulties. For non-adherence to the MD, the prevalence was 26.5% in women without economic difficulties (*μ*₀₀, doubly advantaged), 33.2% in women with economic difficulties (*μ*₀₁), 35.5% in men without economic difficulties (*μ*₁₀) and 42.7% in men with economic difficulties (*μ*₁₁, doubly disadvantaged).

**Figure 3:**
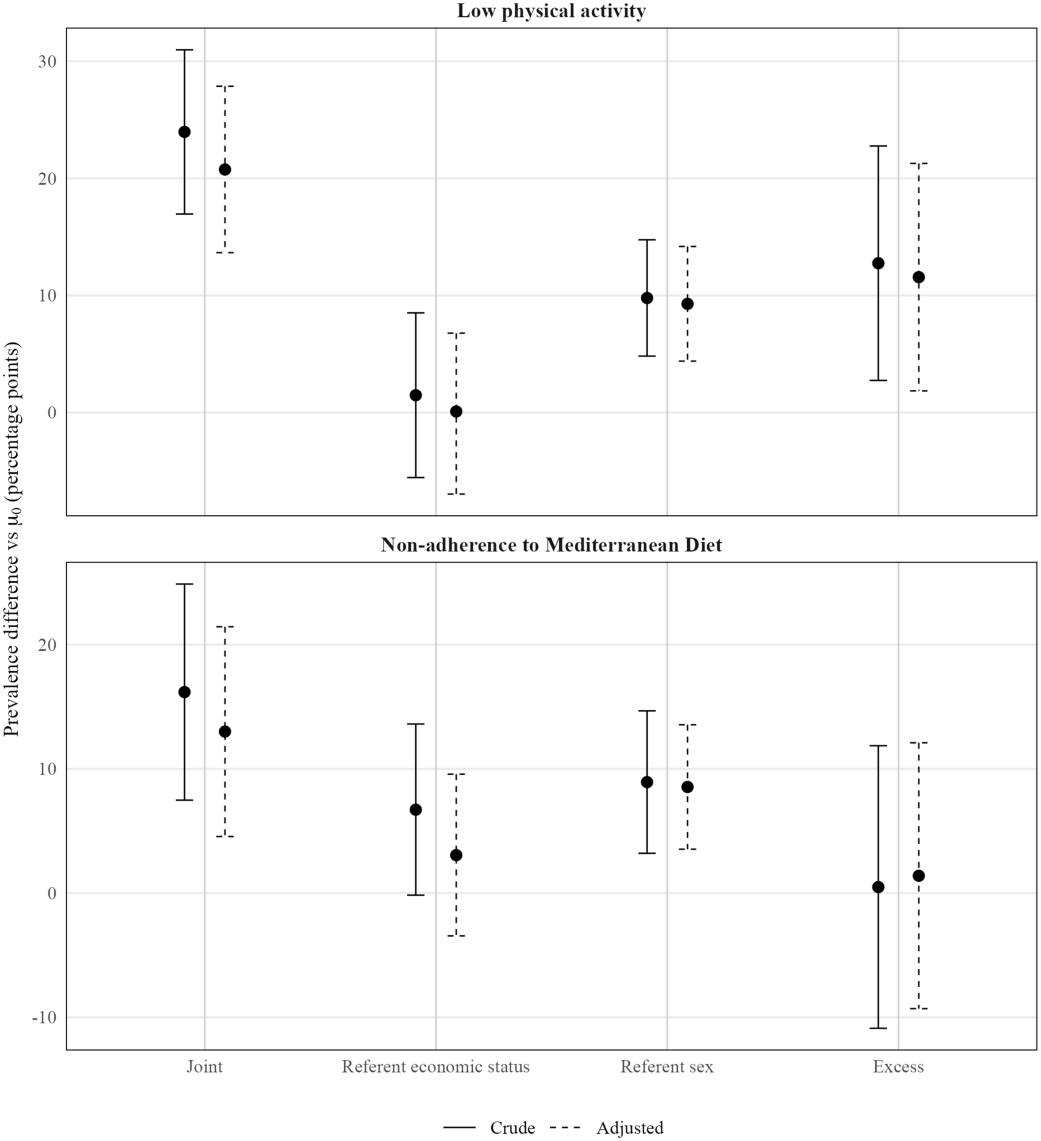
Crude and adjusted prevalence differences (percentage points) in low physical activity (upper panel) and non-adherence to the Mediterranean diet (lower panel) across intersectional inequalities defined by sex and economic difficulties.

Crude and adjusted analyses of disparities led to similar conclusions (Table 4). For low PA, the crude joint disparity between the doubly disadvantaged group (women with economic difficulties, *μ*₁₁) and the doubly advantaged group (men without economic difficulties, *μ*₀₀) was 24.4 percentage points (40.9% vs. 16.5%). The corresponding adjusted joint disparity was 20.0 pp (95% CI 13.6–27.8). In the adjusted decomposition, the referent disparity for economic difficulties (*μ*₀₁ − *μ*₀₀) was small and not statistically significant, whereas the referent disparity for sex (*μ*₁₀ − *μ*₀₀) and the excess intersectional disparity (*μ*₁₁ − *μ*₁₀ − *μ*₀₁ + *μ*₀₀) were substantial. The excess disparity was 11.56 pp (95% CI 1.85–21.27), and the wide confidence interval indicates limited precision for this component, reflecting the smaller size of the doubly disadvantaged group. For non-adherence to the MD, the crude joint disparity between men with economic difficulties (*μ*₁₁) and women without economic difficulties (*μ*₀₀) was 16.2 pp (42.7% vs. 26.5%). In the adjusted analyses, only the referent disparity for sex remained statistically significant (8.54 pp, 95% CI 3.53–13.55), whereas neither the referent disparity for economic difficulties nor the excess intersectional disparity reached statistical significance (Table 4; Figure 3). Table 5 reports the results of the supplemental discriminatory accuracy of the intersectional groups. Although the inclusion of the intersectional groups did not improve substantially the accuracy for both outcomes, the increase was slightly better for the low PA.

**Table 4:** Crude and adjusted inequalities in physical activity practice and adherence to Mediterranean diet.

Inequality	Prevalence difference							
	Low physical activity (METs 1st quartile)				Non-adherence to MD			
	Crude		Adjusted		Crude		Adjusted	
	pp. (95% CI)	*p*	pp. (95% CI)	*p*	pp. (95% CI)	*p*	pp. (95% CI)	*p*
Joint	23.96 (16.95–30.97)	<0.001	20.75 (13.65–27.86)	<0.001	16.18 (7.48–24.88)	<0.001	13.00 (4.55–21.44)	0.003
Referent sex	9.78 (4.82–14.75)	<0.001	9.28 (4.39–14.18)	<0.001	8.98 (3.29–14.67)	0.002	8.54 (3.53–13.55)	0.001
% of joint inequality^1^	40.81		44.72		55.50		65.69	
Referent economic status	1.48 (−5.55–8.51)	0.680	0.09 (−6.96–6.78)	0.979	6.71 (−0.17–13.61)	0.056	3.05 (−3.451–9.57)	0.358
% of joint inequality^1^	6.17		0.43		41.47		23.46	
Excess intersectional inequality	12.75 (2.74–22.76)	0.012	11.56 (1.85–21.27)	0.020	0.48 (−10.89–11.86)	0.933	1.39 (−9.31–12.10)	0.799
% of joint inequality^1^	53.21		55.71		2.96		10.69	

1 % of joint inequality was calculated as (component/joint inequality) × 100 for each component (joint, referent and excess). These percentages are presented as descriptive indicators of the relative contribution of each component and are not additional parameters of the Jackson decomposition.

**Table 5:** Discriminatory accuracy of variables estimated by the area under the receiver operating characteristic curve (AUC^a^).

Model	Non-adherence to MD (95% CI)	Δ AUC vs Model 1	Low physical activity (95% CI)	Δ AUC vs Model 1
Model 1^b^	0.66 (0.63–0.69)	–	0.62 (0.59–0.65)	–
Model 2^c^	0.67 (0.64–0.70)	0.01	0.65 (0.62–0.68)	0.03
Model 3^d^	0.67 (0.64–0.70)	0.01	0.66 (0.63–0.70)	0.04

a AUC values and 95% confidence intervals were used descriptively. Changes in AUC < 0.05 were considered of limited substantive importance and formal statistical tests of differences between AUCs were not performed.

b Model 1: Age + Marital status + Persons in the household + Persons <16 years + Tobacco + Hours/non-working day TV/computer/videogames.

c Model 2: Model 1 + Sex + Economic difficulties.

d Model 3: Model 1 + Intersectional groups.

In the DA analyses (Table 5), the baseline model including only statistically significant covariates (Model 1) showed low-to-moderate ability to distinguish individuals with and without the outcomes, with an AUC of 0.66 for non-adherence to the MD and 0.62 for low PA. Adding sex and economic difficulties as separate predictors (Model 2) produced only small increases in AUC for non-adherence to the MD (ΔAUC = 0.01) and for low PA (ΔAUC = 0.03). Replacing the single axes with the four intersectional groups (Model 3) yielded an AUC of 0.67 (0.64–0.70, ΔAUC = 0.01 vs. Model 1 and ΔAUC = 0.00 vs. Model 2) for non-adherence to the MD and 0.66 (0.63–0.70, ΔAUC = 0.04 vs. Model 1 and ΔAUC = 0.01 vs. Model 2) for low PA. Thus, all AUC values remained around 0.62–0.67 and all changes in AUC were <0.05, indicating low discriminatory capacity and suggesting that intersectional group membership does not meaningfully improve the ability to differentiate individual-level risk.

## 4 Discussion

Our study aimed to estimate population-based inequalities in PA practice and MD intake between groups at the intersection of sex and economic difficulties in northern Spain, and to examine the additional discriminatory accuracy of the intersectional groups. We found joint disparities between the doubly disadvantaged and doubly advantaged positions, of approximately 20 percentage points for low PA and 16 percentage points for non-adherence to the MD. In both outcomes, these inequalities were driven by sex, referent disparities for sex were relevant and statistically significant, whereas referent disparities for economic difficulties were comparatively small and often non-significant. This pattern suggests that, in this Mediterranean context, sex differences in PA and diet remain more pronounced than the socioeconomic gradient captured by our measure of economic difficulties. We also assessed discriminatory accuracy using the area under the ROC curve to evaluate how strongly low PA and non-adherence to the MD were concentrated within specific intersectional groups. AUC values around 0.62–0.67 and very small changes when adding sex, economic difficulties or their intersection indicated low discriminatory capacity, suggesting that these groups do not meaningfully differentiate individual-level risk, despite the presence of sizeable average inequalities.

The contribution of sex to the joint inequality aligns with prior research indicating that women, on average, tend to participate in less PA than men across diverse countries (10). This result can be attributed to the fact that women may encounter more obstacles to engaging in physical exercise activities, including time constraints, limited social support, and concerns related to safety (22). Likewise, studies have shown that the relationship between sex and PA depends on the examined PA domain (23). Regarding the economic status, we observed little evidence of income-related disparities in low PA, the referent disparity for economic difficulties was small and not statistically significant, whereas the referent disparity for sex and the excess disparity were higher. The absence of differences in PA practice associated with economic income identified in this study contrasts with prior research that has demonstrated a general trend wherein individuals with a higher economic status tend to engage in more PA (12). Income inequality may significantly contribute to insufficient PA due to factors such as limitations in infrastructure and access to facilities or resources (12). However, it has been observed that in low-income and socioeconomically disadvantaged communities, the associations between the built environment and PA are weaker and often inverse (24). One possible explanation, which we can only propose as a hypothesis, is that in the province of Girona access to public spaces, walkable environments and community sports facilities may be relatively widespread across economic strata, reducing income-related barriers to PA (25). Furthermore, a comprehensive network of community initiatives, facilitated by primary care centers, actively disseminates and promotes the utilization of these resources (26). This robust infrastructure may be related to the absence of a correlation between economic income and PA engagement in our study. From an intersectional perspective, our decomposition helps clarify how sex and economic difficulties combine to shape inequalities in low PA. The joint disparity compares the doubly disadvantaged group (women with economic difficulties) to the doubly advantaged group (men without economic difficulties) and was approximately 20 percentage points. The referent disparities show that part of this gap can be attributed to sex alone (women vs. men at the same economic level) and a much smaller part to economic difficulties alone (economic difficulties vs. no difficulties within the same sex). The excess disparity, in turn, captures how much worse the doubly disadvantaged group fares than would be expected if the single-axis disparities simply added up. In our data, the excess component corresponds to roughly half of the joint disparity, indicating that the intersection of being a woman and having economic difficulties amplifies the disadvantage in PA beyond the sum of each axis considered separately. Intersectionality theory points out that individuals’ resources, experiences, and identities are shaped not only by specific individual characteristics, such as sex or economic status, but also by the intricate interplay of these diverse characteristics (27). Our results are in agreement with a recent intersectional analysis in a sample of adolescents that suggest that low economic status and female sex are associated with vulnerabilities for low PA practice which may not be separate, but rather interactive and multiplicative in their effects (28).

Regarding adherence to the MD, our study found that the joint inequality achieved a prevalence difference of 16.2 pp. between the doubly advantaged group with respect to the doubly disadvantaged group. Overall, irrespective of the economic status, female groups exhibited a higher adherence to the MD, and sex was responsible for more than 65% of the joint inequality. These results are similar to previous studies that have found better adherence in women (29), but this phenomenon is not generalized, and sex differences in adherence to the MD remain inconclusive (30). It has been suggested that differences between sex may be related to differences in red meat consumption (31), and higher animal protein intake in men (32). Likewise, women tend to consume fruit and vegetable more regularly (33), but other factors such as the body mass index or even food neophobia have been also related to the adherence to the MD (34). In our study, income-related differences in adherence to the Mediterranean diet were modest. The referent disparities associated with economic difficulties did not exceed approximately 5–7 percentage points and were often not statistically significant. This small gradient should be interpreted with caution. First, economic status was measured through a subjective “making ends meet” indicator, which captures perceived financial strain but may smooth finer income gradients and lead to some misclassification. Second, in Mediterranean settings core components of the MD (vegetables, legumes, cereals and olive oil) are culturally embedded and widely available, which may lower cost-related barriers to following the MD compared with other European contexts. If traditional, healthier options remain relatively affordable, socioeconomic differences in MD adherence may be attenuated, while other factors become more salient. Our data cannot directly test these mechanisms, but they provide a plausible explanation for the limited income-related inequalities observed in MD adherence in this region. The current evidence suggests that the adoption of a dietary pattern has been related to numerous factors, including taste, convenience, nutritional value, and cost (35). There is evidence suggesting that adhering to the Mediterranean dietary pattern tends to involve higher costs, and that could be an economic barrier to adopt this dietary pattern (36). However, it has also been observed that countries within Mediterranean-type ecosystems regions with lower socioeconomic status showed higher MD adherence, whereas other European countries in other regions with highest Human Development Index seemed to have lower adherence to the MD (14). Furthermore, it has been suggested that the Mediterranean diet is actually closer to a lifestyle pattern, rather than just a diet that only includes what people eat (37). This lifestyle pattern has been characterized by being physically active, observed mainly in rural areas of the Mediterranean-type ecosystems, and accompanied by low stress levels and activities that are known to reduce psychological stress (38). Considering the study region characteristics, located in northern Catalonia (Spain) and marked by a large number of rural villages and a limited number of cities with more than 10,000 inhabitants (20 out of 221), it is plausible to consider that the adherence to the MD could be more related to lifestyle and that the economic factor may have a limited effect.

The heterogeneity in the outcomes among the intersectional groups can be examined quantifying the differences between strata estimates, but it has been pointed that quantifying the discriminatory accuracy of the intersectional categorization for specific outcomes provides an improved picture of the heterogeneity produced by inequality axes (39). In our study, discriminatory accuracy analyses complemented the mean differences in prevalence by evaluating how strongly low PA and non-adherence to the MD were concentrated within specific intersectional groups. Across all models, AUC values ranged from 0.62 to 0.67 and increased by less than 0.05 when adding sex, economic difficulties or their intersectional combination, indicating low-to-moderate discriminatory capacity. This means that, although intersectional groups differ on average in their risk of low PA and non-adherence to the MD, group membership has limited ability to distinguish which individuals will actually experience these outcomes. This methodological approach can be employed to guide interventions based on the principle of proportionate universalism, and low discriminatory accuracy values would be indicative of universal interventions while high values would be suggestive of a specific interventions (40). In this study, the overall accuracy of the adjusted models could be considered moderate, but the inclusion of the intersectional groups did not provide an additional discriminatory accuracy to the inclusion of both inequality axes separately. Our findings on discriminatory accuracy therefore do not support planning health promotion interventions based solely on intersectional group membership. Nonetheless, joint prevalence differences between doubly advantaged and doubly disadvantaged groups were above 10 percentage points (around 20 pp. for low PA and 16 pp. for non-adherence to the MD), and it has been emphasized that, while the discriminatory accuracy measure serves as a crucial complement to means-centric analyses of health inequalities, its implications for equity-oriented interventions must be interpreted with caution because low AUC values do not undermine the existence of sizeable group-level inequalities, but rather indicate that these inequalities do not translate into strong individual-level prediction (41).

There are limitations that should be considered when interpreting the study results. First, its cross-sectional design precludes causal inference. The temporal ordering between economic difficulties, sex, PA practice and adherence to the MD cannot be established, and reverse causation is possible. For example, poor health status may reduce PA or influence dietary choices, rather than the other way around. Longitudinal studies are needed to determine whether the observed intersectional inequalities translate into future differences in health outcomes. Second, this was a population-based study that used a probabilistic sampling procedure to obtain a high level of external validity. However, the response rate was moderate, and there remains the possibility of some selection bias when generalizing the results. Regarding participants excluded due to missing data on some variables, we did not observe meaningful differences in personal characteristics (Supplementary material). Third, PA is a complex behavior, and opportunities to be active exist in several domains in life. We measured the PA practice by using the Minnesota-Regicor Short Physical Activity Questionnaire for the Adult Population, that is an instrument which allows to capture the total mild–moderate-to-vigorous PA into METs, but does not discriminate the contributions of PA at work or in the household, nor the PA for travel, and during leisure time in a more precise form. Given the reported sex and age differences in domain-specific PA behaviors, we could not rule out some information bias regarding this outcome due to recall or social desirability bias. Using domain-specific and, when feasible, device-based measures of PA would allow a more precise assessment of intersectional inequalities in movement behaviors. Fourth, we measured the economic status based on the Deleeck poverty line approach, an indirect subjective measure, rather than household income or the level of household expenditure/consumption. This methodological approach may have introduced a differential information bias because demographic and socioeconomic characteristics have been related to the subjective perception. Future research should combine subjective indicators of economic hardship with more objective measures of socioeconomic position to better characterize economic inequalities. Among the strengths of this study, it should be noted the population-based approach and the large number of covariates included.

## 5 Conclusion

The use of an intersectional approach has the potential to enhance public health research by offering an improved mapping of socioeconomic health inequalities. Healthy diet may be promoted by universal interventions in Mediterranean-type regions, but study findings suggest that promotion of PA practice may benefit addressing inequalities by the intersection of sex and economic status. In this sense, health promotion interventions should be tailored according to both axes to address the specific needs, barriers, and facilitators of these different subgroups.

## Data Availability

The raw data supporting the conclusions of this article will be made available by the authors without undue reservation.
